# Effects of Continuous and Cycled Annealing on the Physicochemical Properties and Digestibility of Water Caltrop Starch

**DOI:** 10.3390/foods12193551

**Published:** 2023-09-25

**Authors:** Jia-Chen Chung, Lih-Shiuh Lai

**Affiliations:** Department of Food Science and Biotechnology, National Chung Hsing University, 145 Xingda Road, Taichung 40227, Taiwan; frog90814@gmail.com

**Keywords:** water caltrop, annealing, physicochemical properties

## Abstract

The effects of treatment time of continuous annealing (ANN) and cycle numbers of cycled ANN on the structural, physicochemical, and digestive properties of water caltrop starch were studied under 70% moisture at 65 °C. It was found that continuous and cycled ANN have no significant effects on the morphology of starch granules. However, the relative crystallinity and content of resistant starch increased pronouncedly, possibly due to crystalline perfection, which also led to the rise in gelatinization temperature and the narrowed gelatinization temperature range of starch. The treatment time in continuous ANN generally showed a pronounced effect on the rheological properties of water caltrop starch. During pasting, the breakdown viscosity and setback viscosity of all treatment decreased, implying that ANN modified starch was less susceptible to the condition in heating and continuous shearing, and less likely to cause short-term retrogradation. In contrast, peak viscosity decreased with increasing treatment time of continuous ANN, indicating crystalline perfection restricted the swelling of starch granules and viscosity development during pasting process, which was consistent with the results of steady and dynamic rheological evaluation. All ANN-modified samples showed pseudoplastic behavior with weak gel viscoelastic characteristic. Under a total annealing time of 96 h, the pasting and rheological properties of water caltrop starch were essentially less affected by annealing cycle numbers. However, multistage ANN showed stronger resistance to enzyme hydrolysis.

## 1. Introduction

Water caltrop (*Trapa taiwanensis* Nakai) is an annual herbaceous aquatic plant that is suitable for a growing environment with a high temperature and abundant moisture and light. Water caltrop is rich in nutritional value and has been shown to have some physiological benefits, for example, the shell has anti-inflammatory [1] and antibacterial properties [2]. Water caltrop fruit belongs to the category of whole grains, being rich in starch, protein, vitamins B, C, and trace elements, and has a high dietary fiber content [3]. Starch extracted from water caltrop shows higher gelatinization temperature and resistant starch content than those commonly used starch sources, such as wheat, corn, tapioca, and potato starch [4,5,6].

Starch, one of the common sources of carbohydrate intake in humans, is abundant in cereal and rhizome. It is often utilized as a thickener [7], stabilizer [8], and fat replacer [9] in the food industry because of its availability and relatively economic price. However, it has limitations of less thermal stability, retrogradation, and syneresis. To expand the scope of application, different modification methods to overcome the defects of starch are commonly classified into three categories, including chemical, enzymatic, and physical modification [10]. Physical modification acts as a green technology for food applications, which has no effluents containing reagents or by-products discharged to the environment and is less expensive as compared to chemical or enzymatic modifications [11].

Annealing (ANN), a type of physical modification, is generally performed in excess moisture content (≥60% *w*/*w*), under a temperature above the glass transition but below the gelatinization temperature of starch for a period of time from minutes to days. It can be regarded as an eco-friendly and cost-effective process compared with other technologies, such as chemical and enzymatical modification, due to the fact that ANN does not involve the use of chemical reagents [12]. During ANN, the excess water penetrates the soft amorphous regions and improves alignments by providing necessary chain mobility and movements of the double helices throughout annealing [12,13]. It also contributes to limited but reversible swelling of the starch granules, which allows for mobility in the crystalline domains [14]. The influence of annealing on starch depends on the starch source and annealing conditions [15]. Starch, after annealing, generally remains intact as granules, shows a higher gelatinization temperature, a narrower temperature range of crystalline melting, and results in a reduction of starch swelling power and solubility [16].

Previous literature has demonstrated the effects of ANN and cycled ANN on physicochemical and digestive properties of different starches, such as normal and waxy wheat starch [17], potato starch [18], and red azuki bean starch [19]. However, in a study about water caltrop starch, Liu et al. [20] mentioned that the properties of ANN-modified water caltrop starch were mostly similar to those of the native one, possibly due to the generally mild tempering condition (50 °C for 24 h) of ANN. Moreover, there is a dearth of studies on the effects of the treatment time of continuous ANN and cycled ANN of water caltrop starch. We hypothesized that ANN treatment under a temperature closer to the onset gelatinization temperature of water caltrop starch may contribute to significant impacts on the properties of water caltrop starch. Therefore, the objective of this study is to investigate the influences of the treatment time of continuous ANN and cycle numbers of cycled annealing on the morphology and crystalline structure, thermal properties, rheological properties including the pasting, steady shear, and dynamic rheological properties, and the resistant starch content of water caltrop starch under a stronger tempering condition.

## 2. Materials and Methods

### 2.1. Materials

Water caltrop (*Trapa Taiwanensis* Nakai) was harvested in the Xiaying district of Tainan, Taiwan. The resistant starch assay kit was purchased from the Megazyme International Ireland, Co. (Wicklow, Ireland). All chemicals and buffers used were of analytical grade.

### 2.2. Starch Isolation

Water caltrop starch was isolated essentially according to the methods of Liu et al. [20]. Briefly, water caltrop fruits were cut in quarters and crushed with deionized water in equal proportions by using a blender. The starch suspension was sieved, then mixed with twice the amount of 0.1% (*w*/*v*) sodium hydroxide solution. After discarding the brown supernatant, the wet starch sediment was mixed with ten times the amount of distilled water to remove impurities. This washing process was repeated several times until the pH value of the supernatant reached to 7. Afterwards, the starch precipitate was dried, milled, sieved, and stored in plastic containers at room temperature.

### 2.3. Continuous and Cycled Annealing Treatment

Water caltrop starch was annealed according to the methods of Chung et al. and Su et al. with some modification [17,21]. Water caltrop starch was put into a tinplate can (diameter 8 cm, height 5 cm) and the moisture level was kept at 70%. To examine the influence of treatment time during continuous ANN, the sample was sealed, equilibrated overnight in a 4 °C refrigerator, and incubated at 65 °C for 12, 24, 48, and 96 h, marked as ANN 12, ANN 24, ANN 48, and ANN 96, respectively. In addition, to examine the influence of cycle numbers in the cycled ANN treatment, the total heating time was set for 96 h, but was divided into 1, 2, 4, and 8 cycles, with 30 min of cooling at room temperature between each cycle. The cycled ANN samples were then designated as ANN 96 (96 h/cycle, 1 cycle), ANN 48 × 2 (48 h/cycle, 2 cycles), ANN 24 × 4 cycles (24 h/cycle, 4 cycles), and ANN 12 × 8 cycles (12 h/cycle, 8 cycles), respectively. All annealed modified starch was dried, grounded, sieved through a 100-mesh screen, and stored in plastic containers at room temperature.

### 2.4. Scanning Electron Microscopy

Starch samples were attached to a double-sided carbon tape with a platinum film coated on the surface via a metal ion coating instrument (JEC-3000FC, JEOL, Tokyo, Japan), then examined with a scanning electron microscope (JSM-7800F, JEOL, Tokyo, Japan).

### 2.5. Starch Crystalline Structure and Relative Crystallinity

The crystalline structure and relative crystallinity of starch samples were analyzed according to Liu et al. [20]. Samples were placed in a desiccator with a relative humidity of 75% by using saturated sodium chloride solution for 7 days to ensure the stability of the starch structure. Samples were then scanned in the range of 3–50° with a scan step of 0.02° by using a high-resolution X-ray diffractometer (X’Pert Pro MRD, PAnalytical, Almelo, The Netherlands, Holland). Relative crystallinity was calculated by utilizing PeakFit software (v4.1.2, 2007, Systat Software, Inc., Palo Alto, CA, USA).

### 2.6. Starch Thermal Properties

The thermal properties of the starch samples, including the onset temperature (T_o_), peak temperature (T_p_), conclusion temperature (T_c_), and gelatinization enthalpy (ΔH), were measured using a differential scanning calorimeter (DSC 1 STAR^®^ System, Mettler Toledo, Greifensee, Switzerland) [22]. Two milligrams of sample was mixed with 8 mg of deionized water in a 40 μL aluminum crucible (ME-27331, Mettler Toledo, Greifensee, Switzerland). The crucibles were hermetically sealed and placed in a 4 °C refrigerator overnight to reach equilibrium. After the crucible returned to room temperature, it was placed on the left side of the sample holder, while an empty crucible was placed on the right side as a control. The experimental conditions were set to heat from 25 °C to 100 °C with a heating rate of 10 °C/min.

### 2.7. Starch Pasting Properties

A total of 6% starch suspension was prepared with deionized water and placed in a rapid visco analyzer (RVA-Ezi, Newport Scientific Pty. Ltd., Warriewood, Austria) for gelatinization. A typical heating/cooling profiles for starch analysis was adapted [20]. The pasting temperature, peak time, peak viscosity, breakdown viscosity, holding strength, setback viscosity, and final viscosity were determined by analyzing the RVA pasting profiles.

### 2.8. Steady Shear Rheological Properties

The steady shear properties of starch samples were evaluated according to Tsai and Lai [23]. Samples were first gelatinized by using RVA as described in Section 2.7, then a portion of the starch paste was placed on the stage of a rheometer (MCR92, Anton Paar, Graz, Austria), and was sheared with a parallel plate (diameter = 50.00 mm, gap size = 1 mm) measuring system at 25 °C from shear rates of 0.1 to 100 s^−1^. The Herschel–Bulkley model was used to describe the steady shear rheological behavior of water caltrop starch paste:

(1) $$\sigma =\sigma_{0}+K{\left(\dot{\gamma }\right)}^{n},$$

where *σ* and *σ*_0_ are the shear stress and yield stress, respectively; *K* is the consistency index; $\dot{\gamma }$ is the shear rate; and *n* is the flow behavior index.

### 2.9. Dynamic Rheological Properties

The dynamic rheological properties were measured using the same rheometer as described in Section 2.8, and the methods of Marboh and Mahanta were followed [24] with slight modifications. The starch paste was placed on the stage and dynamically sheared with a frequency ranging from 0.1 to 100 rad/s under a strain within the linear viscoelastic range. The storage modulus (G′), loss modulus (G″), and loss tangent (tan δ) of the starch paste samples were determined.

### 2.10. In-Vitro Digestibility

The in vitro digestibility of starch samples was analyzed by using the resistant starch assay kit (K-RSTAR) from Megazyme International Ireland, Co. (Wicklow, Ireland), which essentially determined the hydrolysis of starch by a mixture of α-amylase and amyloglucosidase [20,25]. Starch fractions with different in vitro digestibility were calculated by the following equations [20]:

(2) $$RDS\;(g/100\;g\;sample)={∆E}_{20\;min}\times F\times 90/W$$

(3) $$SDS\;(g/100\;g\;sample)={(∆E}_{120\;min}-{∆E}_{20\;min})\times F\times 90/W$$

(4) $$Very\;SDS\;(g/100\;g\;sample)={(∆E}_{16\;h}-{∆E}_{120\;min})\times F\times 90/W$$

(5) $$RS\;(g/100\;g\;sample)={∆E}_{precipitate}\times F\times 90/W$$

(6) TS (g/100 g sample)=RDS+SDS+Very SDS+RS,

where RDS is a rapidly digestible starch that is calculated based on supernatant collected within 20 min; SDS is a slowly digestible starch that is calculated based on supernatant collected between 20 and 120 min; Very-SDS is a very-slowly digestible starch that is calculated based on supernatant collected between 120 min and 16 h; RS is a resistant starch that is calculated based on the precipitate; TS is the total starch content of the samples. ΔE is the absorbance (reaction) read against the reagent blank; *F* is the conversion from absorbance to micrograms; and W is the weight of the sample in gram.

### 2.11. Statistical Analysis

The data were analyzed with one-way analysis of variance (ANOVA) followed by Duncan’s multiple range test at a significance level of 95% (*p* < 0.05) by using IBM SPSS Statistics 20 software.

## 3. Results and Discussion

### 3.1. Starch Morphology

**Figure 1:**
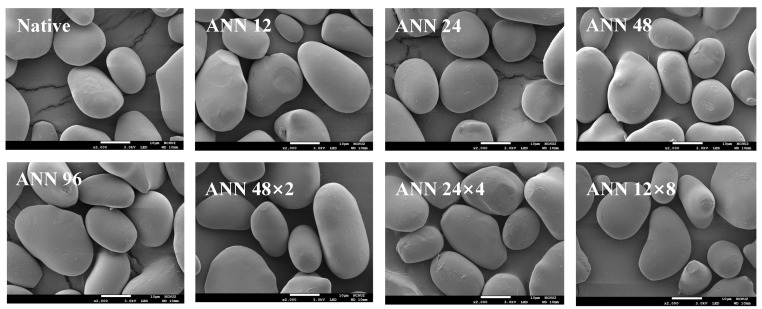
Scanning electron microscopic images of continuous ANN and cycled ANN treated water caltrop starch (2000×). ANN represents annealing treatment; 12, 24, 48 and 96 indicate the duration of continuous annealing treatment in hours. 48 × 2, 24 × 4 and 12 × 8 indicate a total annealing duration of 96 h that is divided into 2, 4, and 8 cycles, respectively.

Figure 1 showed the scanning electron microscope (SEM) images of water caltrop starch after continuous ANN and cycled ANN. It was observed that native water caltrop starch showed an oval and irregular shape. Various continuous and cycled ANN treatments did not cause great change on the surface microstructure of the starch granules, which remained intact. The result was consistent with the findings in the literature, which generally inferred that there was no significant difference in the appearance of starch from different sources and structures after annealing [18,26,27], such as yam starch under an annealing condition at 50 °C for 24 h [26]. Likewise, in a study of potato starch, which had a B-type starch crystalline structure, it was reported that continuous and cycled annealing treatment at 55 °C for up to 96 h did not cause a significant change in the shape of starch granules, except that only part of the starch granules was dented [18]. Moreover, in a study of mung bean starch, which had a C-type starch crystalline structure, it was reported that continuous and cycled annealing treatments at 50 °C for up to 96 h did not change the surface microstructure of starch granules, except that the shallow groove on surface became deeper [27].

### 3.2. Crystalline Properties

**Figure 2:**
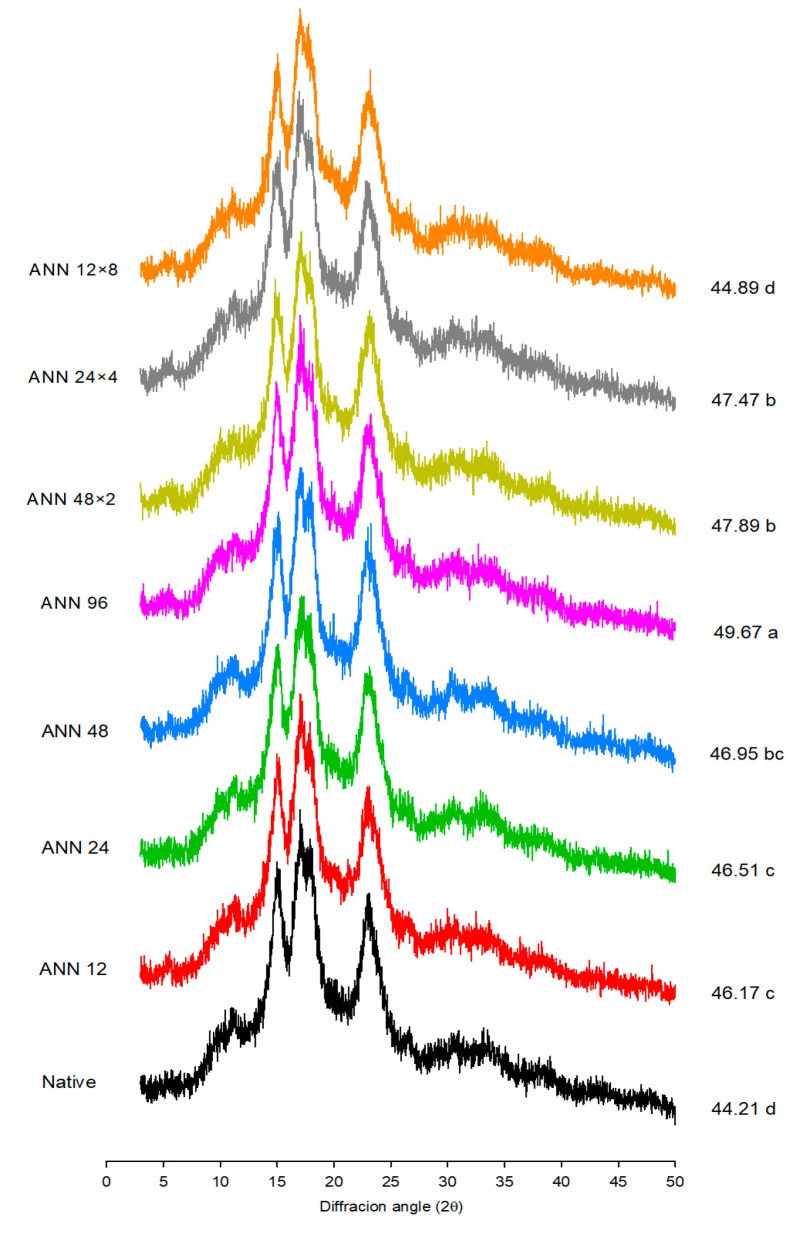
X-ray diffraction pattern and relative crystallinity of continuous ANN and cycled ANN treated water caltrop starch. ANN represents annealing treatment; 12, 24, 48 and 96 indicate the duration of continuous annealing treatment in hours; 48 × 2, 24 × 4 and 12 × 8 indicate a total annealing duration of 96 h that is divided into 2, 4 and 8 cycles, respectively. ^a–d^ Relative crystallinity values, which are labelled on the right for each diffraction pattern, with different letters are significantly different (*p* < 0.05).

X-ray diffraction is used to observe the long-range order associated with the packing of double helices into ordered crystalline structures of starch [28]. The results of the X-ray pattern of native, annealed, and cycle-annealed water caltrop starch are shown in Figure 2. Native water caltrop starch exhibited an XRD pattern that can be characterized as an A-type starch by the presence of strong peaks at 2θ of 15, 17, 18, and 23°. However, after annealing and cycled annealing treatments, the water caltrop starch crystalline structure turned to a C_A_-type structure as evidenced by the appearance of a small peak at 2θ of 5.6°, which was the characteristic peak of B-type polymorphs. In other words, both continuous and cycled ANN treatments led to a partial polymorphic transition from A-type water caltrop starch to B-type, and annealed water caltrop starch can be classified as a C_A_-type polymorph. It was also reported that annealing treatment tends to increase the B-type polymorphs within C-type pea starch and C_A_-type pinhao starch [29,30].

The relative crystallinity of water caltrop starch increased with the increasing treatment time of continuous ANN. The increase in crystallinity can be attributed to the more efficient packing of double helices in the crystalline region [31] due to promoted movement and combination of starch chains. The internal rearrangement as well as the formation of a more ordered structure led to crystalline perfection [32]. Under the same total tempering time of 96 h, it was found that less cycled annealing treatment contributed to a more stable structure, which was similar to the two-stage annealing of rice starch [33] and starch from *Castanopsis sclerophylla* under annealing conditions for one- and two-step annealing [34]. After multi-stage annealing, starch was less conducive to the transformation from an imperfect to a perfect crystalline structure, thereby inhibiting the formation of new microcrystalline structures. Though cycled ANN could improve the packing of microcrystalline and enhance the integrity of the crystal structure, it also led to the expansion of the amorphous regions within starch granules due to the combination of thermal energy and water. As the cycle numbers increased, sufficient stress was generated as the temperature increased, leading to the dissociation of the double helix within the crystalline structure and causing a reduction in crystallinity by multi-stage annealing [35].

### 3.3. Thermal Properties

**Figure 3:**
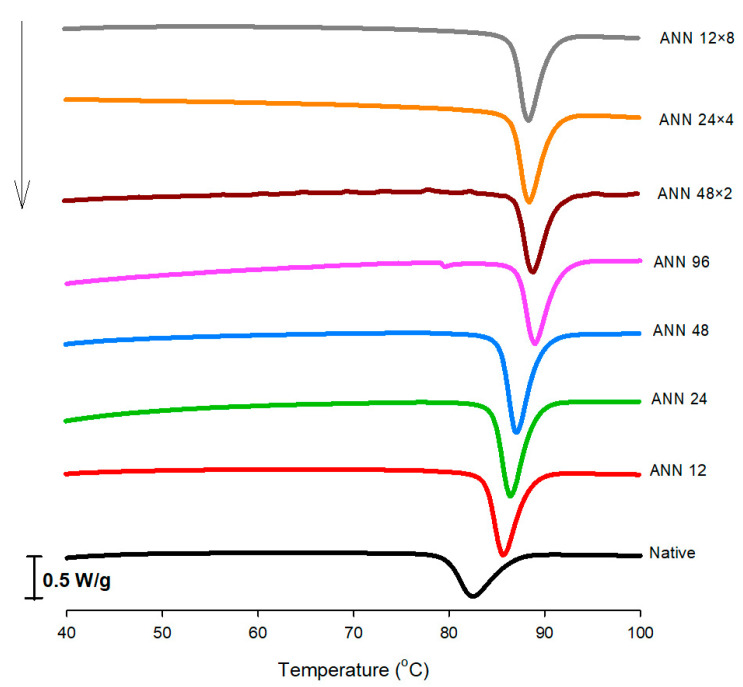
DSC thermograms of continuous ANN and cycled ANN treated water caltrop starch. ANN represents annealing treatment; 12, 24, 48 and 96 indicate the duration of continuous annealing treatment in hours; 48 × 2, 24 × 4 and 12 × 8 indicate a total annealing duration of 96 h that is divided into 2, 4 and 8 cycles, respectively.

Differential scanning calorimetry (DSC) was used to analyze the thermal characteristics of starch gelatinization transition. As shown in Figure 3, the phase-transition-related endothermic changes started to occur at lower temperatures with a wider temperature range for native water caltrop starch. For continuous ANN treatment, as the annealing time increased, the onset for phase transition started at progressively lower temperatures, accompanied by a narrower phase transition temperature range. However, the numbers of annealing cycles did not impart a significant effect on peak transition temperature.

The onset (T_o_), peak (T_p_), and conclusion (T_c_) temperature, as well as the transition enthalpy (ΔH), for native and various annealed water caltrop starches during gelatinization transition are summarized in Table 1. As shown in Table 1, all transition temperatures, including onset, peak, and conclusion temperatures, increased with the increasing treatment time of continuous ANN. These results demonstrated that the gelatinization transition of the imperfect starch microcrystalline structure was significantly affected by annealing, and a longer annealing time can further improve the weaker crystalline structure [36]. The higher gelatinization temperature caused by annealing is possibly owing to the perfection of a less stable crystalline structure, enhanced interaction between amylose/amylose and amylose/amylopectin, and increased ordering of amorphous regions by annealing treatment [26]. The results were consistent with the findings shown in Figure 2, also confirming that the rearrangement of starch chains may form a more compact crystalline structure, which requires higher energy for gelatinization. In addition, the reduction of the gelatinization temperature range (T_c_–T_o_) implied that annealing can promote the formation of homogeneous microcrystalline and crystalline stability [28,37]. Under a total annealing time of 96 h, cycle numbers generally did not affect the gelatinization transition significantly, which was in line with studies on sweet potato starch under cycled annealing conditions [38].

ΔH is related to the enthalpy for starch gelatinization and is also associated with the energy of the double helix in amylopectin disintegration [39]. Table 1 shows that continuous ANN-modified water caltrop starch required a higher ΔH for phase transition with increasing annealing time, which was attributed to an improvement in double helix registration and the lengthening of double helices through ordering of the unordered ends of double helices [40]. The increase of ΔH proved that annealing had a great effect on making crystalline structure more stable, and the prolonged ANN treatment further facilitated the crystalline structure perfection. However, under a total annealing time of 96 h, the numbers of annealing cycles did not show a definite trend on changing ΔH, possibly due to a combined controversial effect of thermal energy and water during multi-stage annealing [35].

### 3.4. Pasting Properties

The results on the pasting properties of water caltrop starch after continuous and cycled ANN are shown in Table 2. The peak time and pasting temperature of water caltrop starch significantly increased with increasing treatment time of continuous ANN, which was possibly related to the strengthening of bonds and enhancement of the interaction between amylose and amylopectin within starch granules [41]. However, the numbers of annealing cycles did not impart a significant effect. Compared with native water caltrop starch, all ANN-modified starch showed a lower viscosity because annealing intensified the interaction between starch chains and formed more compact crystalline as shown in Figure 2, which contributed to limiting starch swelling and the reduction of leaching amylose content. Breakdown viscosity is the difference between peak viscosity and trough viscosity, standing for the resistance to heat and shear force. A decrease in breakdown viscosity through annealing was observed, implying that ANN-modified starch was less susceptible to the conditions of heating and continuous shearing. Setback viscosity is the subtraction of holding strength from final viscosity, reflecting starch retrogradation. It was found that all ANN-modified starch was less likely to cause short-term retrogradation. However, the numbers of annealing cycles showed less impact on pasting properties, which was consistent with the findings for wheat starch [42].

**Figure 4:**
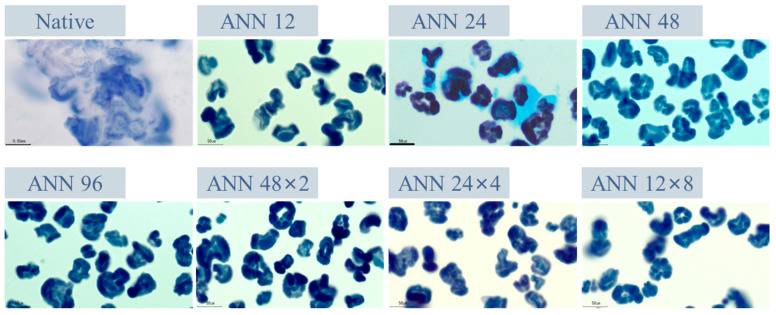
Iodine-staining images of continuous ANN and cycled ANN treated water caltrop starch pastes. ANN represents annealing treatment; 12, 24, 48 and 96 indicate the duration of continuous annealing treatment in hours; 48 × 2, 24 × 4 and 12 × 8 indicate a total annealing duration of 96 h that is divided into 2, 4, and 8 cycles, respectively.

Figure 4 exhibits the iodine-staining morphology of native, continuous ANN and cycled ANN modified starch paste. It demonstrated that the starch granules of native water caltrop starch were essentially destroyed after pasting in RVA, accompanied with significant leaching-out of starch molecules. In contrast, the appearance of all ANN-modified starch paste still retained the intact starch granular borderline, indicating that crystalline perfection restricted the swelling and viscosity development of starch granules during the pasting process [43].

### 3.5. Steady Shear Rheological Properties

**Figure 5:**
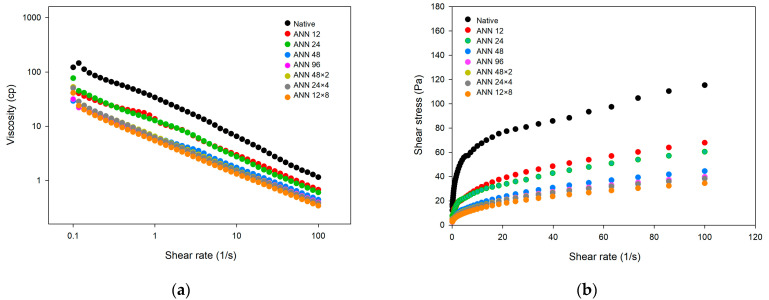
Steady shear rheo-grams of continuous ANN and cycled ANN treated water caltrop starch. (**a**) Viscosity versus shear rate. (**b**) Shear stress versus shear rate. ANN represents annealing treatment; 12, 24, 48 and 96 indicate the duration of continuous annealing treatment in hours; 48 × 2, 24 × 4 and 12 × 8 indicate a total annealing duration of 96 h that is divided into 2, 4, and 8 cycles, respectively.

The steady shear properties of water caltrop starch after continuous and cycled ANN treatment are demonstrated in Figure 5. With increasing shear rate, the viscosity of native and all ANN-modified water caltrop starch decreased, indicating that water caltrop starch paste is a shear-thinning fluid. Moreover, native water caltrop starch showed high static yield stress, which was possibly attributed to weak gel formation from short-term starch retrogradation. After continuous and cycled ANN modification, the yield stress of water caltrop starch reduced significantly, possibly because annealing inhibited starch swelling that influenced viscosity development.

Table 3 presented the rheological parameter of water caltrop starch paste by using the Herschel–Bulkley model. In terms of the effect of treatment time of continuous ANN, the flow behavior index (n) slightly increased; however, consistency index (K) decreased after annealing modification due to restricted amylose leaching and lowered swelling power, which hindered the short-term retrogradation. Moreover, under a total annealing time of 96 h, cycle numbers of cycled ANN showed less impact on the steady shear rheological properties, which was essentially in line with the pasting properties shown in Table 2.

Furthermore, in the dietary design for patients with dysphagia, a shear rate of 50 s^−1^ has been adopted as the standard by which the viscosity of food is measured for dysphagia management [44]. According to the classification guide of National Dysphagia Diet, the results in Table 3 showed that the viscosity of native water caltrop starch at a shear rate of 50 s^−1^ was 1.846 Pa∙s (=1846 cP), indicating a pudding-like texture. After undergoing various annealing modification, the viscosity decreased to 0.540–0.969 Pa∙s (=540–969 cP), resulting in a honey-like texture [45]. However, it should be mentioned that η_50_ (viscosity at 50 s^−1^) in relation to dysphagic diets may not cover the entire range of shear rate variations during the swallowing process. Our results showed that the value of η_50_ could only differentiate the textures between native water caltrop starch and annealed water caltrop starch. The difference in viscosity (Table 2) and K values (Table 3) among different ANN treatment groups could not be distinguished by the value of η_50_. Therefore, connecting various rheological data with sensory perceptions remains challenging.

### 3.6. Dynamic Rheological Properties

**Figure 6:**
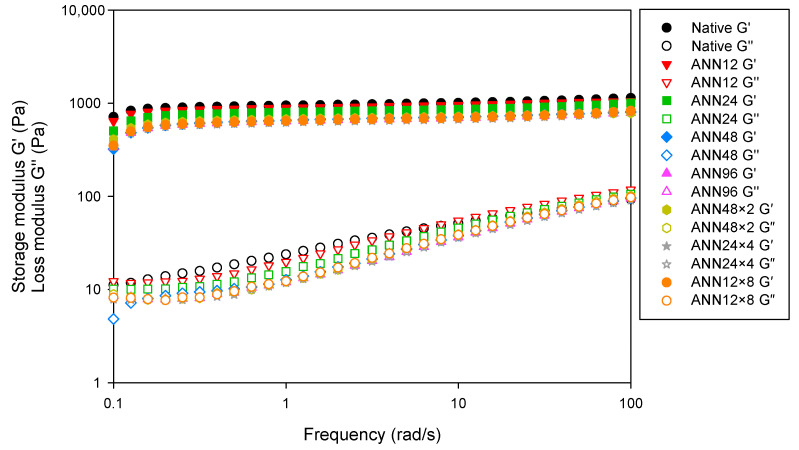
Dynamic frequency sweep rheo-grams of continuous ANN and cycled ANN treated water caltrop starch. ANN represents annealing treatment; 12, 24, 48 and 96 indicate the duration of continuous annealing treatment in hours; 48 × 2, 24 × 4 and 12 × 8 indicate a total annealing duration of 96 h that is divided into 2, 4, and 8 cycles, respectively.

In an oscillatory rheological assessment, storage modulus (G′) represents elastic and solid-like properties, and loss modulus (G″) refers to viscous and liquid-like properties. Figure 6 displayed the frequency dependence of G′ and G″ for native and ANN modified starch. Native water caltrop starch showed the highest G′ and G″, possibly due to starch gel formation by amylose leaching and rearrangement. With increasing the annealing time of continuous ANN, G′ and G″ slightly decreased, implying that the more intact and stable starch structure due to enhanced internal chains resulted in lower swelling power and dynamic modulus [46]. In addition, it was found that the G′ was higher than G″ over the entire frequency range studied, indicating that all water caltrop starch pastes were considered as elastic solids rheologically. When deformation increased, the network structure of water caltrop starch pastes gradually collapsed, leading to significant slope change in G″ over the frequency range studied to weak gel structure [47]. However, under a total tempering time of 96 h, cycle numbers of cycled ANN showed less impact on the dynamic shear rheological properties.

Table 4 summarized the dynamic viscoelastic parameters of water caltrop starch under a frequency of 1 Hz. Both the G′ and G″ decreased with the increasing treatment time of continuous ANN. However, the decrease in G′ is more pronounced as compared to G″. Tan δ is the ratio of G″ to G′, which acts as the ratio of viscous to elastic properties. The declined tan δ after annealing treatment may be attributed to not fully hydrated starch granules, which avoided the disintegration of starch granules during gelatinization by annealing treatment. Generally speaking, the viscoelastic properties of water caltrop starch were less affected by the cycle numbers during cycled ANN treatment.

### 3.7. In-Vitro Digestibility

Based on the in vitro digestibility determined through the hydrolysis of starch by a mixture of α-amylase and amyloglucosidase, starch can be classified into rapid digestible starch (RDS, completely digested within 20 min), slowly digestible starch (SDS, digested between 20 and 120 min), very-slowly digestible starch (Very-SDS, digested between 120 min and 16 h), and resistant starch (RS, not fully digested in 16 h) [20]. Though much previous literature has demonstrated the effects of ANN on resistant starch content, the outcome actually depends on the starch source and annealing conditions [14,17,18,19,20,24,27,33,38]. Table 5 showed the effect of continuous and cycled ANN treatments on the in vitro digestibility of water caltrop starch. It was observed that RDS generally increased after various ANN treatments, possibly because the starch sample has to be ground again after ANN treatment, leading to higher damaged starch content which was more susceptible to enzyme action. However, as the treatment time of continuous ANN or the cycle numbers of cycled ANN increased, a slight decrease in SDS and significant increase in the sum of Very-SDS and RS were observed. SDS is primarily composed of a small amount of double helix crystalline regions and partially ordered amorphous regions [48]. It was believed that a portion of SDS may be turned into Very-SDS and RS as the annealing treatment time or cycle numbers increased. Longer annealing times led to interactions and the rearrangement of double helices, resulting in more uniform crystal packing which prevented water from entering the glycosidic bonds, making it more difficult for enzymes to penetrate and hydrolyze the granules internally [11,49]. As a result, the sum of Very-SDS and RS increased from about 76% to 80%. Moreover, under a total annealing time of 96 h, though the pasting and rheological properties of water caltrop starch were essentially less affected by annealing cycle numbers (Table 2, Table 3, Table 4 and Table 5), the susceptibility to enzyme hydrolysis were significantly altered. The sum of Very-SDS and RS content can be further increased to up to about 83%. In addition, the increase in RS content is more effective for cycled ANN, particularly for sample ANN 48 × 2. These results suggested that the continuous and cycled ANN conditions applied in this study can produce modified water caltrop starch with lower digestibility, which may be beneficial as ingredients in food products for people who have concerns about controlling blood sugar.

## 4. Conclusions

This study showed that both the treatment time of continuous and the cycle number of cycled ANN had a pronounced impact on the relative crystallinity and starch compositions based in-vitro digestibility, particularly the sum of very-slow digestible starch and resistant starch (RS) content. These results suggest that the continuous and cycled ANN conditions applied in this study, particularly ANN 48 × 2, can produce modified water caltrop starch with lower digestibility, which may be beneficial for people who have concerns about controlling blood sugar level. Moreover, the continuous and cycled ANN conditions applied in this study pronouncedly improved the thermal stability, shear resistance, and resistant starch contents of water caltrop starch due to crystalline perfection, which enhanced the application range of water caltrop starch. The impact of the treatment time of continuous ANN on the properties of water caltrop starch was generally greater than that of the cycle numbers of cycled ANN. In contrast, the increase in RS content is more effective for cycled ANN. However, the in vitro digestibility, determined through the hydrolysis of starch by a mixture of α-amylase and amyloglucosidase, cannot completely represent the exact digestion in vivo, and may also be altered during processing. Therefore, more research on real systems is needed for a better understanding of the effect of processing and in vivo conditions.

## Figures and Tables

**Table 1:** Differential scanning calorimetric parameters of continuous ANN and cycled ANN treated water caltrop starch ^1–3^.

Sample	T_o_ (°C)	T_p_ (°C)	T_c_ (°C)	T_c_–T_o_ (°C)	ΔH (J/g)
Native	79.76 ± 0.25 ^d^	82.36 ± 0.35 ^d^	86.49 ± 0.25 ^d^	6.74 ± 0.02 ^a^	14.89 ± 0.21 ^d^
ANN 12	84.08 ± 0.53 ^c^	85.90 ± 0.53 ^c^	88.73 ± 0.59 ^c^	4.66 ± 0.06 ^b^	16.45 ± 0.04 ^bc^
ANN 24	84.52 ± 0.03 ^bc^	86.18 ± 0.02 ^c^	88.76 ± 0.13 ^c^	4.24 ± 0.10 ^c^	16.75 ± 0.32 ^b^
ANN 48	85.25 ± 0.01 ^b^	86.94 ± 0.12 ^b^	89.40 ± 0.01 ^c^	4.15 ± 0.01 ^c^	17.23 ± 0.06 ^a^
ANN 96	86.63 ± 0.70 ^a^	88.26 ± 0.70 ^a^	91.64 ± 0.93 ^a^	4.10 ± 0.45 ^c^	16.19 ± 0.20 ^c^
ANN 48 × 2	86.78 ± 0.40 ^a^	88.42 ± 0.35 ^a^	91.04 ± 0.38 ^ab^	4.26 ± 0.05 ^c^	15.33 ± 0.44 ^d^
ANN 24 × 4	86.41 ± 0.22 ^a^	88.07 ± 0.21 ^a^	90.63 ± 0.35 ^b^	4.21 ± 0.13 ^c^	16.42 ± 0.04 ^bc^
ANN 12 × 8	86.52 ± 0.28 ^a^	88.16 ± 0.27 ^a^	90.60 ± 0.24 ^b^	4.08 ± 0.08 ^c^	16.03 ± 0.07 ^c^

^1^ T_o_, T_p_, and T_c_ represent onset, peak, and conclusion temperature, respectively. T_c_–T_o_ represents gelatinization temperature range. ΔH represents gelatinization enthalpy. ^2^ Data are expressed as mean ± standard deviation (*n* = 3). Values in the same column with different letters are significantly different (*p* < 0.05). ^3^ ANN represents annealing treatment; 12, 24, 48 and 96 indicate the duration of continuous annealing treatment in hours; 48 × 2, 24 × 4 and 12 × 8 indicate a total annealing duration of 96 h that is divided into 2, 4, and 8 cycles, respectively.

**Table 2:** Rapid-visco parameters of continuous ANN and cycled ANN treated water caltrop starch ^1,2^.

Sample	Peak Time (min)	Pasting Temperature (°C)	Peak Viscosity (cp)	Breakdown (cp)	Holding Strength (cp)	Setback (cp)	Final Viscosity (cp)
Native	4.82 ± 0.05 ^b^	85.23 ± 0.30 ^d^	1057.33 ± 31.34 ^a^	81.67 ± 27.43 ^a^	975.67 ± 20.21 ^a^	461.00 ± 20.00 ^a^	1436.67 ± 33.29 ^a^
ANN 12	5.17 ± 0.00 ^a^	88.72 ± 0.46 ^c^	383.67 ± 12.50 ^b^	−169.00 ± 5.57 ^b^	552.67 ± 7.23 ^b^	221.67 ± 3.06 ^b^	777.67 ± 10.97 ^b^
ANN 24	5.17 ± 0.00 ^a^	88.83 ± 0.03 ^c^	279.00 ± 6.00 ^c^	−166.00 ± 4.58 ^b^	445.00 ± 6.24 ^c^	194.00 ± 3.00 ^c^	639.00 ± 7.55 ^c^
ANN 48	5.16 ± 0.06 ^a^	89.65 ± 0.54 ^b^	216.67 ± 8.14 ^d^	−159.00 ± 6.08 ^b^	375.67 ± 2.08 ^d^	175.67 ± 5.51 ^d^	551.33 ± 7.51 ^d^
ANN 96	5.19 ± 0.02 ^a^	90.12 ± 0.03 ^ab^	193.33 ± 7.64 ^de^	−155.67 ± 5.03 ^b^	349.00 ± 2.65 ^e^	163.33 ± 1.53 ^d^	512.33 ± 3.51 ^e^
ANN 48 × 2	5.16 ± 0.05 ^a^	90.27 ± 0.20 ^a^	175.33 ± 6.03 ^e^	−149.67 ± 3.21 ^b^	325.33 ± 5.86 ^fg^	164.33 ± 2.31 ^d^	489.67 ± 5.51 ^ef^
ANN 24 × 4	5.13 ± 0.05 ^a^	90.43 ± 0.35 ^a^	176.67 ± 8.96 ^e^	−163.67 ± 3.79 ^b^	340.33 ± 7.51 ^ef^	164.33 ± 3.21 ^d^	504.67 ± 10.26 ^ef^
ANN 12 × 8	5.17 ± 0.00 ^a^	90.57 ± 0.10 ^a^	168.00 ± 13.00 ^e^	−149.00 ± 3.46 ^b^	317.00 ± 16.09 ^g^	161.67 ± 2.31 ^d^	478.67 ± 18.23 ^f^

^1^ Data are expressed as mean ± standard deviation (*n* = 3). Values in the same column with different letters are significantly different (*p* < 0.05). ^2^ ANN represents annealing treatment; 12, 24, 48 and 96 indicate the duration of continuous annealing treatment in hours; 48 × 2, 24 × 4 and 12 × 8 indicate a total annealing duration of 96 h that is divided into 2, 4 and 8 cycles, respectively.

**Table 3:** Herschel–Bulkley rheological parameters of continuous ANN and cycled ANN-treated water caltrop starch ^1–3^.

Sample	Herschel-Bulkley				
	σ_0_ (Pa)	η_50_ (Pa∙s)	n	K (Pa∙s^n^)	R^2^
Native	14.255 ^a^	1.846 ^a^	0.372 ^a^	22.408 ^a^	0.986
ANN 12	4.022 ^b^	0.969 ^b^	0.406 ^a^	9.723 ^b^	0.998
ANN 24	3.772 ^b^	0.838 ^b^	0.405 ^a^	8.244 ^bc^	0.999
ANN 48	2.217 ^b^	0.667 ^c^	0.408 ^a^	6.665 ^cd^	0.999
ANN 96	1.337 ^b^	0.546 ^c^	0.430 ^a^	5.081 ^d^	0.999
ANN 48 × 2	2.316 ^b^	0.566 ^c^	0.406 ^a^	5.675 ^d^	0.999
ANN 24 × 4	1.797 ^b^	0.547 ^c^	0.436 ^a^	4.899 ^d^	0.999
ANN 12 × 8	2.128 ^b^	0.540 ^c^	0.415 ^a^	5.262 ^d^	0.999

^1^ σ_0_ represents yield stress, η_50_ represents the viscosity at the shear rate of 50 s^−1^, K represents consistency index, and the R^2^ represents coefficient of determination. ^2^ Data are expressed as mean ± standard deviation (*n* = 3). Values in the same column with different letters are significantly different (*p* < 0.05). ^3^ ANN represents annealing treatment; 12, 24, 48 and 96 indicate the duration of continuous annealing treatment in hours; 48 × 2, 24 × 4 and 12 × 8 indicate a total annealing duration of 96 h that is divided into 2, 4, and 8 cycles, respectively.

**Table 4:** Dynamic viscoelastic parameters of continuous ANN and cycled ANN treated water caltrop starch (frequency = 1 Hz) ^1,2^.

	G′ (Pa)	G″ (Pa)	tan δ
Native	974.420 ^a^	46.655 ^a^	0.048 ^a^
ANN 12	928.775 ^b^	43.882 ^a^	0.047 ^ab^
ANN 24	838.545 ^c^	36.739 ^b^	0.044 ^abc^
ANN 48	706.480 ^d^	30.774 ^c^	0.044 ^abc^
ANN 96	692.415 ^d^	29.002 ^c^	0.042 ^c^
ANN 48 × 2	674.335 ^de^	28.414 ^c^	0.043 ^bc^
ANN 24 × 4	654.155 ^e^	27.592 ^c^	0.042 ^c^
ANN 12 × 8	694.600 ^d^	29.548 ^c^	0.043 ^bc^

^1^ Data are expressed as mean ± standard deviation (*n* = 3). Values in the same column with different letters are significantly different (*p* < 0.05). ^2^ ANN represents annealing treatment; 12, 24, 48 and 96 indicate the duration of continuous annealing treatment in hours; 48 × 2, 24 × 4 and 12 × 8 indicate a total annealing duration of 96 h is divided into 2, 4, and 8 cycles, respectively.

**Table 5:** Starch compositions of continuous ANN and cycled ANN treated water caltrop starch based on in vitro digestibility ^1,2^.

	RDS (<20 min) (g/100 g Sample)	SDS (20–120 min) (g/100 g Sample)	Very-SDS (120 min–16 h) (g/100 g Sample)	Resistant Starch (>16 h) (g/100 g Sample)	Total Starch (g/100 g Sample)
Native	3.81 ± 0.03 ^cd^	10.75 ± 0.19 ^a^	38.63 ± 0.17 ^d^	37.52 ± 0.30 ^e^	90.72 ± 0.62 ^b^
ANN 12	4.83 ± 0.01 ^ab^	7.84 ± 0.01 ^c^	41.41 ± 0.01 ^a^	37.88 ± 0.74 ^de^	91.96 ± 0.94 ^ab^
ANN 24	3.39 ± 0.22 ^d^	9.50 ± 0.56 ^b^	40.34 ± 0.33 ^bc^	38.63 ± 0.65 ^cd^	91.87 ± 0.47 ^ab^
ANN 48	5.06 ± 0.62 ^ab^	8.11 ± 0.65 ^c^	39.43 ± 1.01 ^cd^	39.44 ± 0.21 ^c^	92.42 ± 1.33 ^a^
ANN 96	4.28 ± 0.26 ^bc^	7.08 ± 0.04 ^cd^	41.39 ± 0.01 ^a^	39.30 ± 0.31 ^c^	91.89 ± 0.21 ^ab^
ANN 48 × 2	5.42 ± 0.37 ^a^	5.78 ± 0.19 ^e^	35.53 ± 0.17 ^e^	46.92 ± 0.63 ^a^	93.38 ± 0.22 ^a^
ANN 24 × 4	5.16 ± 0.42 ^a^	6.26 ± 1.13 ^de^	34.73 ± 0.71 ^f^	42.62 ± 0.59 ^b^	88.04 ± 0.89 ^c^
ANN 12 × 8	5.24 ± 0.32 ^a^	5.28 ± 0.41 ^e^	40.70 ± 0.12 ^ab^	42.14 ± 0.08 ^b^	93.36 ± 0.48 ^a^

^1^ Data are expressed as mean ± standard deviation (*n* = 3). Values in the same column with different letters are significantly different (*p* < 0.05). ^2^ ANN represents annealing treatment; 12, 24, 48 and 96 indicate the duration of continuous annealing treatment in hours; 48 × 2, 24 × 4 and 12 × 8 indicate a total annealing duration of 96 h that is divided into 2, 4, and 8 cycles, respectively.

## Data Availability

The data presented in this study are available on request from the corresponding author. The data are not publicly available due to ethical restriction and the intellectual property issue.
